# Epilepsy care guidelines for low- and middle- income countries: From WHO mental health GAP to national programs

**DOI:** 10.1186/1741-7015-10-107

**Published:** 2012-09-24

**Authors:** Juri Katchanov, Gretchen L Birbeck

**Affiliations:** 1 Department of Infectious Diseases and Gastroenterology, Auguste-Viktoria-Krankenhaus, Berlin, Germany; 2 Queen Elizabeth Central Hospital, Blantyre, Malawi; 3 Michigan State University, International Neurologic and Psychiatric Epidemiology Program, East Lansing, Michigan, USA; 4 Chikankata Hospital, Epilepsy Care Team, Mazabuka, Zambia

**Keywords:** Non-physician healthcare worker, clinical officer, variations in care, treatment gap

## Abstract

In 2011, the World Health Organization's (WHO) mental health Gap Action Programme (mhGAP) released evidence-based epilepsy-care guidelines for use in low and middle income countries (LAMICs). From a geographical, sociocultural, and political perspective, LAMICs represent a heterogenous group with significant differences in the epidemiology, etiology, and perceptions of epilepsy. Successful implementation of the guidelines requires local adaptation for use within individual countries. For effective implementation and sustainability, the sense of ownership and empowerment must be transferred from the global health authorities to the local people. Sociocultural and financial barriers that impede the implementation of the guidelines should be identified and ameliorated. Impact assessment and program revisions should be planned and a budget allocated to them. If effectively implemented, as intended, at the primary-care level, the mhGAP guidelines have the potential to facilitate a substantial reduction in the epilepsy treatment gap and improve the quality of epilepsy care in resource-limited settings.

## Review

### Introduction

Epilepsy is a major global healthcare issue. An estimated 70 million people worldwide live with epilepsy [1,2], most of whom remain untreated [3]. To improve epilepsy-care services, most developed countries adopted national guidelines at least a decade ago, with these being regularly updated [4-6]. Whenever possible, such guidelines are evidence-based, relying upon the body of evidence for best care practices in high-income, resource-rich countries with predominantly moderate climates. However, 80% of people with epilepsy (PWE) live in so-called 'developing', low-income, or resource-poor countries in tropical or subtropical regions[1]. For many reasons including resource restrictions, simply adopting healthcare guidelines created for higher-resourced areas and using these in resource-limited settings is neither appropriate nor feasible [7]. Unfortunately, such settings generally lack sufficient neurologic expertise and/or advocacy for the development and implementation of epilepsy-care guidelines. The World Health Organization (WHO) recently released evidence-based, epilepsy-care guidelines appropriate for use in resource-limited, tropical settings [8,9]. Implementation of the WHO mental health Gap Action Programme (mhGAP) guidelines will require local adaptation for use within individual countries, but if effectively implemented as intended at the primary-care level, the mhGAP guidelines could facilitate a substantial reduction in the epilepsy treatment gap and improve the quality of care received by PWE in resource-limited settings. Effective guidelines require local adaptation, implementation, impact assessment, and program revision. The challenges to these important processes are substantial.

### World Health Organization Mental Health Gap Action Programme guideline development

Low-income and high-income regions differ substantially in terms of the underlying etiologies of epilepsy, the expertise of the healthcare personnel, and the diagnostic capacity, treatment options, and cultural perception of the disorder (Table 1). Guidelines for use in the resource-poor environments must address factors specific for the clinical and sociocultural setting (Table 2). Valid guidelines are of paramount importance for low-income tropical regions as the mortality associated with epilepsy in such regions may be relatively high even when there is treatment available [10], and the treatment gap remains vast, with less than 20% of people with active epilepsy in most developing countries receiving treatment [11].

**Table 1:** Critical differences affecting health and health care in high-income versus low-income and middle-income countries [19,23]

	High-income countries	Low-income and middle-income countries
Gross national income per capita	High ≥US$9,386; upper middle US$3,036 to $9,385	Low ≤US$765 or lower; middle US$766 to $3,035

Access to health care	Initial access usually through primary care with established referral networks, which may include high indirect costs	Limited to very basic primary care especially in rural areas and/or established referral networks, which invariably include high indirect costs

Healthcare funding	National programs, private insurance, out-of-pocket expenses	Often ill-funded, may rely on donors/volunteering services. Indirect costs and informal payments can represent major barriers to care

Common epilepsy etiologies	Neoplastic, cerebrovascular	(Post-)infectious, antenatal, post-traumatic

HIV prevalence	Low	Can be moderate to high

Cultural perception of seizures	Biomedical model	Traditional medicine, spiritual approach; contagion beliefs common

Socio-cultural attitudes towards epilepsy	Neutral public perception or at least social presentation of neutrality	Overt negative public perception, stigmatization, and discrimination common

**Table 2:** Realities and requirements for guidelines for low-income and middle- income countries

Reality	Requirement
Care is largely provided by non-physician healthcare workers with very basic or no neurological training	Clear case definition of epileptic seizures and simple algorithms tailored for the local circumstances

Limited access to medication	Guidelines recommending those medications that can be accessed

Indirect costs as a barrier to care-seeking and adherence	Priority for inexpensive affordable drugs delivered as close to the patient's residence as possible

High prevalence of infectious causes	Incorporate into guidelines testing/treating of common conditions such as HIV, neurotuberculosis, and parasitosis. Refer to existing treatment guidelines whenever possible unless comorbid conditions require care that differs from national guidelines

Guidelines developed in high-income countries are likely to be inappropriate for use in LAMICs [7]. In general, the transferability of evidence derived from studies conducted in high- income countries to LAMICS is dubious [7,12,13]. Differences in patient populations and healthcare systems are so prominent that the evidence may not be valid [7,14]; hence, the development of guidelines specifically crafted for resource-limited settings is the best strategy.

In 1977, the WHO, in collaboration with the International League against Epilepsy and the International Bureau for Epilepsy, launched the Global Campaign Against

Epilepsy to improve the care of PWE in resource-poor countries [15]. In 2008, WHO began development of evidence-based guidelines for epilepsy and seizure care in LAMICs [8,16], and these guidelines were released in 2011.

### Local adaptation of the guidelines

The clinical care algorithms provided in the mhGAP were developed for use in a wide range of possible low-income and middle- income settings, and therefore must be adapted to local resources and needs, especially if the guidelines are to be used by the non-physician healthcare workers who provide most primary healthcare services in such settings. From the geographical, sociocultural, and political viewpoints, countries previously termed 'developing' represent a markedly heterogenous group. The epilepsy burden is different in Asia, Latin America, and sub-Saharan Africa [17], and even within one area, such as sub-Saharan Africa, there are significant variations in the epidemiology, etiologies, and perceptions of epilepsy in different geographical regions and communities [18]. The process of adaption also offers the opportunity to further foster a sense of ownership and empowerment among local health authorities [19].

The most feasible and cost-effective way to deliver epilepsy care in LAMICs is through the use of inexpensive antiepileptic drugs (AEDs) delivered by non-physician healthcare workers at the primary-care level [20]. Clinical case definitions for epileptic seizures and epilepsy used for guideline application must consider the limited neurologic expertise of primary-care providers, lack of diagnostic options, and the local syntactic/semantic language used for describing seizure symptoms, as well as the time frame of the symptoms [21]. The mhGAP guidelines recognize that in LAMICs, seizures are often caused by acute central nervous system (CNS) infection or metabolic disorders. Furthermore, epilepsy can be the first presentation of a sub-acute or chronic CNS condition that might be amenable to treatment in resource-poor environment [20]. Hence, local adaptation of mhGAP guidelines must consider the local epidemiology of potential underlying seizure etiologies [20,22].

Local adaptation must also address any special circumstances within a specific country. For example, where HIV rates are high, a significant proportion of PWE can be expected to also have co-morbid HIV infection. If the available AEDs are limited to enzyme-inducing agents, potential interactions between AEDs and antiretroviral medications must be considered, and treatment options appropriate for dual therapy must be made available [23,24]. Guidelines for epilepsy care in resource-limited settings by non-physician healthcare workers must also specifically address injury prevention and, if applicable, fears of contagion [10]. The adapted content of such programs must be directed by local practices, injury risk factors, and beliefs.

The development of national programs in LAMIC should be paralleled by clinical research increasing our knowledge of epilepsy beyond high-income regions. Such research would facilitate the development of a new multidimensional classification of epilepsy applicable to a wide range of settings including LAMIC [25,26].

### Implementing guidelines in low-income and middle- income countries

Passive dissemination of guidelines is on its own insufficient to ensure appropriate uptake of recommendations [27]. Sociocultural and financial barriers impede the implementation of guidelines in all healthcare settings. Within LAMICs, strong advocacy for guideline adoption by health authorities at the national, provincial, district, and institutional levels are required [27]. Barriers at the patient, healthcare worker and macro level threaten the implementation of guidelines [23]. True advocacy requires that local healthcare authorities prioritize epilepsy care sufficiently highly to guarantee that the basic materials and training required to adhere to the guidelines are provided to healthcare workers at every level of care for which they are intended. If care equity is to be achieved, special attention must be focused on the implementation of the guidelines in poorer rural areas, because residency in a rural region is an independent risk factor for poor access to treatment [3].

Particularly in rural areas, 'buy-in' from local stakeholders is of paramount importance. This requires seeking active input from local communities in developing national priorities and programs at an early stage. Such stakeholders should also be involved the program-evaluation process. Important local stakeholders may include individuals such as traditional healers, who are often the *de facto *care providers for PWE [28]. Whenever possible, collaborations that do not compromise patient care and safety should be sought. Provoking a culture clash between traditional and 'western-type' medicine is unlikely to benefit the patient with epilepsy.

### Impact assessment

As guidelines are being implemented, program evaluations to assess their operational performance in clinical practice and their effects on care quality should be concurrently planned and budgeted [29]. As in more medically developed countries [30], low-income countries need valid quality indicators for epilepsy care that can adequately assess the effects of guideline implementation. It may be possible to develop such quality indicators even with basic health records in some LAMIC institutions [31]. Before initial implementation, plans should be discussed for updating guidelines as needed and revising them based upon the findings of the impact assessment. Special consideration should also be given to any potential unintended consequences of the guidelines. For example, if a guideline refers to additional diagnostic studies, is it possible for primary-care workers to delay necessary seizure treatment while waiting for an electroencephalogram or neuroimaging, when access to such studies are limited and the logistics of study acquisition are challenging?

## Conclusion

**Figure 1:**
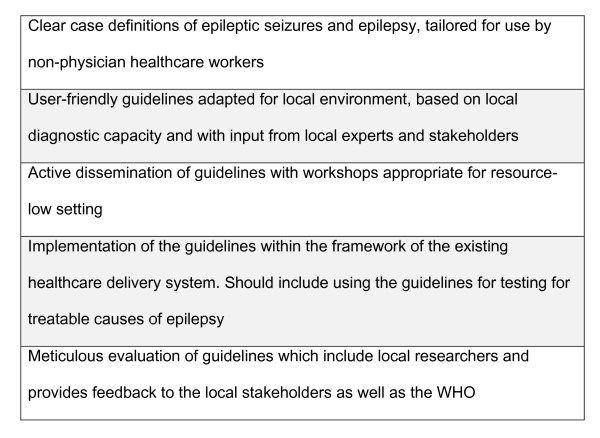
**Primary principles for developing guidelines for epilepsy care in low-income countries**.

The mhGAP guidelines need to be adapted for country-specific use in LAMICs. These baseline recommendations can facilitate the development of national guidelines and the establishment of national epilepsy programs tailored to the existing healthcare setting. The locally relevant guidelines should then be critically evaluated and amended if necessary, based on the results of the assessment (Figure 1). Evaluations are needed to ensure that the guidelines are practical, evidence-based. and cost-effective [32,33]. Training resources (including sample pathways and video training) adapted for local primary-care settings should be expanded to facilitate the acceptance and successful implementation of the guidelines [9]. The implementation of epilepsy guidelines could result in a decrease in the burden of epilepsy worldwide [16].

## Abbreviations

AED: antiepileptic drug; CNS: central nervous system; LAMIC: low- and middle- income countries; mhGAP: mental health Gap Action Programme; PWE: people with epilepsy; WHO: World Health Organization.

## Competing interests

JK declares no conflict of interest or competing interests. GB served as an advisor to the World Health Organization and participated in the development of the mental health Gap Action Programme guidelines and the National Guidelines for Epilepsy Care adopted by the Neurologic and Psychiatric Society of Zambia. She has received research funds for epilepsy-related work from the US National Institute of Health.

## Authors' Information

GLB and JK are neurologists who trained in academic centers in the developed world, but they have extensive experience providing epilepsy care in tropical, resource-limited settings.

## Authors' contributions

GLB and JK co-conceived the idea for this commentary. JK sought relevant literature for this manuscript and wrote the first draft of the manuscript. GLB provided critical input and edits for the final version. Both authors approved the final manuscript.

## Pre-publication history

The pre-publication history for this paper can be accessed here:

http://www.biomedcentral.com/1741-7015/10/107/prepub
